# Association Between Galactose-Deficient IgA1 Derived From the Tonsils and Recurrence of IgA Nephropathy in Patients Who Underwent Kidney Transplantation

**DOI:** 10.3389/fimmu.2020.02068

**Published:** 2020-09-03

**Authors:** Mayuko Kawabe, Izumi Yamamoto, Takafumi Yamakawa, Haruki Katsumata, Nao Isaka, Ai Katsuma, Yasuyuki Nakada, Akimitsu Kobayashi, Kentaro Koike, Hiroyuki Ueda, Yudo Tanno, Yusuke Koike, Jun Miki, Hiroki Yamada, Takahiro Kimura, Ichiro Ohkido, Nobuo Tsuboi, Hiroyasu Yamamoto, Hiromi Kojima, Takashi Yokoo

**Affiliations:** ^1^Division of Nephrology and Hypertension, Department of Internal Medicine, The Jikei University School of Medicine, Tokyo, Japan; ^2^Department of Otorhinolaryngology, The Jikei University School of Medicine, Tokyo, Japan; ^3^Department of Urology, The Jikei University School of Medicine, Tokyo, Japan

**Keywords:** IgA nephropathy, tonsillectomy, galactose-deficient IgA1, recurrent glomerulonephritis, kidney transplantation

## Abstract

**Background:** Recurrence of IgA nephropathy (IgAN) in the transplanted kidney is associated with graft survival, but no specific treatment is available. Tonsillectomy (TE) reportedly arrests the progression of IgAN in the native kidney. Thus, we conducted a single-center retrospective cohort study to evaluate the effect of TE prior to IgAN recurrence.

**Methods:** Of the 36 patients with biopsy-proven IgAN who underwent kidney transplantation, 27 were included in this study. Nine patients underwent TE at 1 year after kidney transplantation (group 1), and the remaining 18 did not undergo TE (group 2).

**Results:** The rate of histological IgAN recurrence was significantly lower in group 1 than in group 2 (11.1 vs. 55.6%, log-rank *p* = 0.046). In addition, half of the recurrent patients in group 2 exhibited active lesions, compared to none in group 1. Serum Gd-IgA1 levels decreased after TE in group 1, whereas they remained stable or increased slightly in group 2. In the recurrent cases, IgA and Gd-IgA1 were found in the germinal center in addition to the mantle zone of tonsils. Finally, mesangial IgA and Gd-IgA1 immunoreactivity was reduced after TE in some cases.

**Conclusion:** Our data suggest that TE at 1 year after kidney transplantation might be associated with the reduced rate of histological IgAN recurrence. TE arrested or reduced serum Gd-IgA1 and mesangial Gd-IgA1 immunoreactivity. Therefore, we generated a hypothesis that serum Gd-IgA1 derived from the tonsils may play a pivotal role in the pathogenesis of IgAN. Based on these findings, we need to conduct verification in a prospective randomized controlled trial.

## Introduction

IgA nephropathy (IgAN) is the most frequent form of glomerulonephritis worldwide, and accounts for approximately half of all primary glomerular diseases diagnosed by kidney biopsy. IgAN progresses to end-stage kidney disease (ESKD) in 10–20% of patients at 10 years and in 20–40% at ~20 years after diagnosis (1). Kidney transplantation is effective for patients with ESKD; however, glomerulonephritis can recur after transplantation, and such recurrent glomerulonephritis, especially focal segmental glomerulosclerosis, membranoproliferative glomerulonephritis, and IgAN are strongly associated with a higher risk of graft failure (2, 3). With regard to the pathogenesis of IgAN, the most widely accepted pathogenesis is “four-hit” hypothesis, including production of Galactose-deficient IgA1 (Gd-IgA1), IgG or IgA autoantibodies that recognize Gd-IgA1, and their subsequent immune complexes formation and glomerular deposition (4). Recently, the abnormality of tonsil or intestinal mucosal immunity have received attention as the core of IgAN immunopathogenesis (5). In Asian cohort, many studies have reported that tonsillectomy (TE) was associated with an improved kidney survival rate and a recent study suggested that the timing of TE in a short window before and after steroid pulse therapy was very important for the effect on IgAN (6). In our institution, we perform TE for IgAN recurrence after kidney transplantation; the outcomes are favorable without immunosuppressive therapy (7, 8). However, there was little data about the effective timing of TE in kidney transplantation.

In this study, we hypothesized TE prior to relapse could associate the reduction rate of IgAN recurrence and evaluated the effect in patients who did and did not undergo TE at 1 year after kidney transplantation. In addition, we evaluated pathological features (in tonsils and allograft biopsies), and serum Gd-IgA1 levels pre- and post-TE.

## Materials and Methods

### Patients

**Figure 1:**
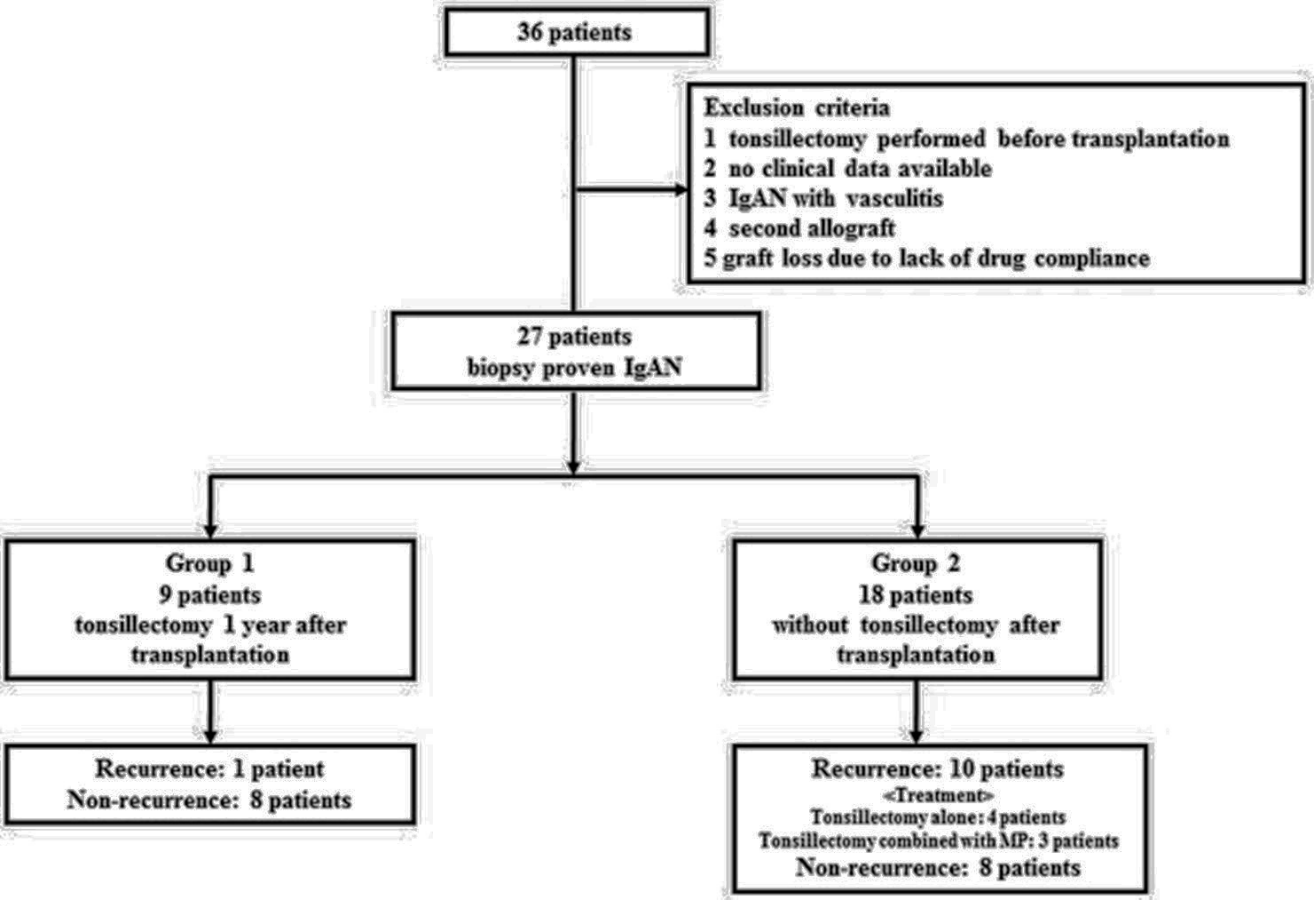
Subject enrolment flow chart. From May 1988 to June 2014, 36 patients with biopsy-proven IgA nephropathy (IgAN) underwent kidney transplantation in our hospital. Among the 36 patients, we excluded those with the following: tonsillectomy (TE) performed before transplantation, no clinical data available, IgAN with vasculitis, a second allograft, and graft loss due to lack of drug compliance. In total, 27 patients were included in this study. Among them, nine underwent TE at 1 year after kidney transplantation (group 1), and the remaining 18 did not undergo TE after transplantation (group 2).

Thirty-six patients with ESKD and biopsy-proven primary IgAN received kidney transplants at the Jikei University School of Medicine (Tokyo, Japan) from May 1988 to June 2014. Some cases were involved in previous studies using the same database (9, 10). All patients received allografts from living related and unrelated donors. Nine patients were excluded from the study as four underwent TE before transplantation, clinical data were missing for two after transplantation, one had concurrent vasculitis, one received a second allograft, and one lost the allograft due to non-compliance with the immunosuppressant therapy. Of the remaining 27 patients, nine (group 1) underwent TE at 1 year after kidney transplantation and 18 (group 2) did not (Figure 1). Histological and clinical data were collected from the time of transplantation until May of 2018. To evaluate the effect of TE on clinical parameters, 5-year post-transplantation serum creatinine levels, proteinuria status, and hematuria status were analyzed. Written informed consent was obtained from all individual participants for the publication of any potentially identifiable images or data included in this article.

### Criteria of Tonsillectomy

Based on the many reports that TE was effective for the treatment of IgAN, we hypothesized that TE prior to relapse could suppress IgAN recurrence after kidney transplantation. All patients provided informed consent for TE. TE was an invasive surgery, so we explained the risk to the patients and performed TE at 1 year after kidney transplantation in which renal function was stabilized in the cases with consent obtained. In addition, as in the past, TE was performed as the treatment for IgAN recurrence even in cases which TE was not selected at 1 year after kidney transplantation. The tonsils obtained from patients with sleep apnoea syndrome (SAS) and native IgAN were subjected to pathological analysis. This study was performed in accordance with the Declaration of Helsinki and approved by the Ethics Committee of the Jikei University School of Medicine.

### Episodic Biopsy Criteria and Definition of Histological/Clinical IgA Recurrence

Allograft biopsies were performed as protocol and/or episodic biopsies. A protocol biopsy was performed routinely at 3 months and at 1, 3, and 5 years after kidney transplantation. We also performed a 0-h biopsy during the operation to exclude transmitted mesangial IgA deposition (or IgA nephropathy) from the donor. In our institution, episodic biopsies are performed in patients who meet the following criteria: persistent proteinuria >0.5 g/day, decreased renal function, and a serum creatinine level that is 1.5-fold that at baseline or >0.3 mg/dL. The biopsies were evaluated using light microscopy and immunofluorescence. Histological recurrence of IgAN was defined as positive immunofluorescence staining for IgA in the mesangial area. Biopsies were evaluated according to the 2016 Oxford classification of IgA nephropathy; (11) the criteria are identical to those for the diagnosis of primary IgAN. We also defined clinical IgA recurrence as histological IgAN recurrence in an episodic biopsy. All biopsy specimens from kidney allograft recipients were evaluated according to the 2017 Banff classification.

### Measurement of Serum Gd-IgA1 Levels

To evaluate the effect of TE, serum samples were collected at the time of kidney transplantation and post- TE or post-transplantation. The serum samples were stored at −80°C prior to use. Serum Gd-IgA1 levels were measured using KM55 sandwich enzyme-linked immunosorbent assay (ELISA) kits (#27600, Immuno-Biological Laboratories) in accordance with the manufacturer's instructions after dilution in ELISA buffer (1:250). We compared serum Gd-IgA1 levels between the two groups and according to histological recurrence.

### Double Immunostaining for IgA and Gd-IgA1

IgA and Gd-IgA1 deposition in allograft biopsy specimens and tonsil tissues was examined using immunofluorescence staining. Paraffin-embedded sections of 4-μm thickness were prepared for staining. After deparaffinisation in a PathoClean®/ethanol series and rehydration, antigen retrieval with subtilisin A (P5380, Sigma-Aldrich) was performed at room temperature for 2 h. Next, samples were blocked with non-fat dry milk (#9999S, Cell Signaling Technology) at room temperature for 60 min, followed by incubation with KM55 (1:10 dilution; #10777, Immuno-Biological Laboratories) at 4°C overnight. After several washes with phosphate-buffered saline, an Alexa Fluor 568-conjugated goat anti-rat IgG (1:200 dilution; Life Technologies) was added at room temperature for 60 min followed by incubation with fluorescein isothiocyanate-conjugated polyclonal rabbit anti-human IgA (1:50 dilution; Dako) at 37°C for 30 min. Fluorescence was observed using a BZ-X800 microscope (Keyence).

### Statistical Analysis

Results are expressed as the means ± standard deviation. Statistical comparisons between the two groups were conducted using the Mann–Whitney *U*-test. Patient and graft survival rates were compared using Fisher's exact test and the log-rank test. Statistical analysis was performed using Stata ver. 14.0 software (StataCorp). A *p* < 0.05 was considered indicative of statistical significance.

## Results

### Baseline Characteristics of the Patients

The clinical characteristics of nine patients (group 1) who received TE at 1 year after kidney transplantation and 18 patients (group 2) who did not receive TE after transplantation are shown in Table 1. The median post-transplant follow-up duration was 9.28 ± 4.11 years for group 1 and 10.9 ± 6.67 years for group 2 (*p* = 0.72). No significant differences in age at transplant, sex, body mass index (BMI), donor age, donor sex, living related donors, ABO blood type incompatibility rate, or zero-human leukocyte antigen (HLA) mismatch were observed between the two groups. All patients received allografts from living donors and most donors were living related donors (26/27; 96.3%). There was only one patient with zero-HLA mismatch. Age at initial diagnosis was 24.7 ± 7.35 years in group 1 and 25.6 ± 12.4 years in group 2 (*p* = 0.82), and the interval from initial diagnosis to dialysis was 10.6 ± 6.37 years in group 1 and 9.69 ± 6.16 years in group 2 (*p* = 0.80). All patients received three immunosuppressive therapies, and most received tacrolimus, mycophenolate mofetil, and prednisolone during the maintenance period. In Japan, anti-thymocyte globulin is not covered by health insurance for kidney transplant recipients, and so was not used. In group 2, IgA deposits were found in the 0-h biopsies of two patients; diminished IgA deposits were then found in both 1-year protocol biopsies. There were no significant differences in hypertension or diabetes after kidney transplantation between the two groups (*p* = 0.18, 0.58).

**Table 1:** Demographic characteristics of the patients.

	**Group 1 with tonsillectomy (*n* = 9)**	**Group 2 without tonsillectomy (*n* = 18)**	***p-*value**
Age at transplant (years)	36.0 ± 4.97	36.9 ± 10.3	0.86
Sex male, *n* (%)	5 (55.6)	11 (61.1)	0.89
BMI (kg/m^2^)	22.7 ± 4.5	20.6 ± 2.9	0.30
Living related donors, *n* (%)	9 (100)	17 (94.4)	1.00
ABO incompatible, *n* (%)	3 (33.3)	9 (50.0)	0.68
HLA class I mismatch	1.22 ± 0.83	1.59 ± 1.00	0.29
HLA class II mismatch	0.56 ± 0.53	0.71 ± 0.59	0.55
Zero-HLA mismatch, *n* (%)	0 (0)	1 (5.6)	1.00
Donor age (years)	57.3 ± 10.0	59.2 ± 7.77	0.62
Donor sex male, *n* (%)	1 (11.1)	6 (33.3)	0.36
Age at native diagnosis (years)	24.7 ± 7.35	25.6 ± 12.4	0.82
Length of native diagnosis to dialysis (years)	10.6 ± 6.37	9.69 ± 6.16	0.80
Length of dialysis (months)	19.7 ± 16.2	33.4 ± 41.7	0.35
Hemodialysis, *n* (%)	4 (44.4)	7 (38.9)	0.89
Follow-up post transplantation (years)	9.28 ± 4.11	10.9 ± 6.67	0.72
Incidence of transmitted mesangial IgA deposition, *n* (%)	0/9 (0)	2/13 (15.4)	0.49
IgA (mg/dL)	246 ± 66.1	212 ± 71.3	0.18
ACEi/ARB, *n* (%)	5 (55.6)	10 (55.6)	0.68
PSL/FK506/MMF, *n* (%)	8 (88.9)	14 (77.8)	0.64
Hypertension, *n* (%)	5 (55.6)	15 (83.3)	0.18
Diabetes, *n* (%)	2 (22.2)	2 (11.1)	0.58

*BMI, body mass index; ACEi, angiotensin-converting enzyme inhibitor; ARB, angiotensin receptor blocker; PSL, prednisolone; MMF, mycophenolate mofetil*.

### Tonsillectomy Complications

No complications occurred during TE in either group.

### IgAN Recurrence

**Figure 2:**
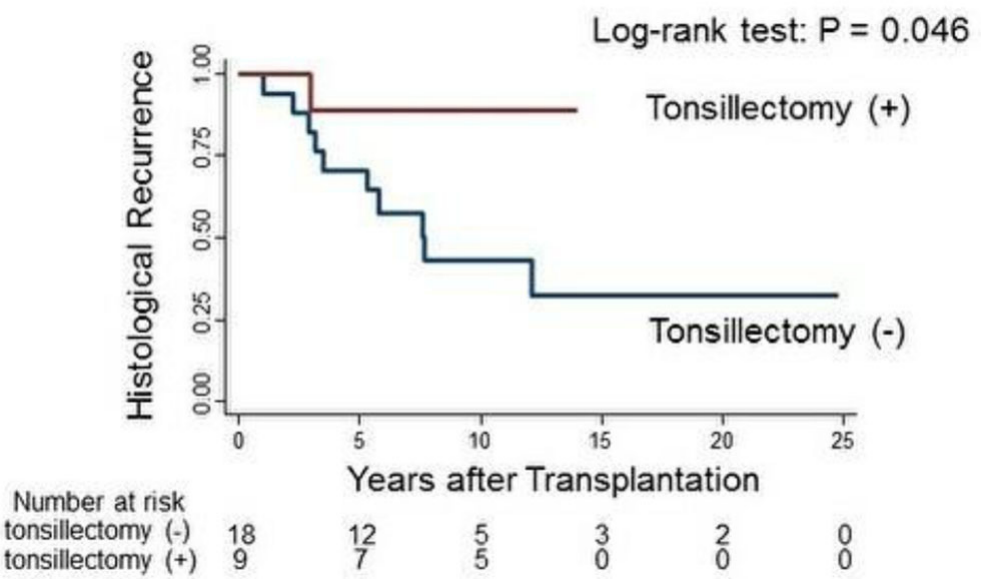
Log-rank test of histological IgAN recurrence in group 1 (red line) and group 2 (blue line).

IgAN recurrence was diagnosed in 41% (11/27) of the patients at 5.0 ± 3.2 years. The histological recurrence rate was 11.1% (1/9 patients) in group 1 and 55.6% (10/18 patients) in group 2 (*p* = 0.04; Table 2). TE at 1 year after kidney transplantation was associated with a significantly reduced rate of histological recurrence of IgAN (*p* = 0.046; Figure 2). There was no clinical recurrence of IgAN in group 1, whereas seven patients in group 2 (38.9%) experienced clinical recurrence (*p* = 0.06). In group 2, only three patients were diagnosed with chronic rejection at the time of clinical IgAN recurrence.

**Table 2:** Comparative study of acute and chronic rejection, graft survival, recurrence, and clinical findings between Group 1 and 2.

	**Group 1 with tonsillectomy (*n* = 9)**	**Group 2 without tonsillectomy (*n* = 18)**	***p-*value**
Acute rejection, *n* (%)	0 (0)	3 (16.7)	0.53
Chronic rejection, *n* (%)	1 (11.1)	6 (33.3)	0.36
Graft survival, *n* (%)	9 (100)	12 (66.7)	0.07
Survival, *n* (%)	9 (100)	17 (94.4)	1.00
Histological recurrence of IgAN, *n* (%)	1/9 (11.1)	10/18 (55.6)	0.04
Clinical recurrence of IgAN, *n* (%)	0/9 (0)	7/18 (38.9)	0.06
Serum creatinine (mg/dL)^*^	1.55 ± 1.20	1.75 ± 0.75	0.15
Proteinuria (mg/day)^*^	260.9 ± 377.5	665.7 ± 822.4	0.16
Hematuria (%)^*^			0.12
Negative or ± 1+ 2+	7/7 (100)	9/15 (60) 4/15 (26.7) 2/15 (13.3)	

*IgAN, IgA nephropathy*.

* *The data of serum creatinine, proteinuria and hematuria were the 5-year post transplantation data*.

### Therapy for IgAN Recurrence and Outcomes

We observed 11 cases of IgAN recurrence (one in group 1 and 10 in group 2). Recurrence with mild segmental proliferation (M0E0S0T0C0 by the 2016 Oxford classification) was observed in the 3-year protocol biopsy of the patient in group 1, who did not exhibit clinical manifestations during follow-up. In group 2, clinical recurrence (diagnosed by episodic biopsies) was observed in seven patients, whereas three patients who lacked clinical features were diagnosed using protocol biopsies (Table 3). Among the seven clinical IgAN recurrence cases, five (71.4%) were associated with high histological activity such as endocapillary and extracapillary proliferation. We treated these patients with three consecutive methylprednisolone pulses combined with TE (three patients), a methylprednisolone pulse alone (one patient), and angiotensin receptor blockers (ARBs) alone (one patient). Four of five patients with histological activity lost graft function after recurrence, not because of IgAN recurrence but due to cABMR. The other two patients who experienced clinical IgAN recurrence without histological activity underwent only TE. Similarly, two of three patients diagnosed via protocol biopsies also underwent only TE. Interestingly, in patient 8, mesangial proliferation improved markedly (Table 3, **Figure 6**); in this patient, segmental mesangial proliferation in <50% of glomeruli and decreased mesangial IgA deposits after TE was observed in the 4-year episodic biopsy. We did not experience allograft loss due to IgAN recurrence during the follow-up period.

**Table 3:** Patients with recurrence of IgA nephrology.

**No**	**Age (years)**	**Time of recurrence (years)**	**Bx**	**Oxford classification**	**S-Cr (mg/dl)**	**Proteinuria (g/day)**	**U-occ (/HPF)**	**Therapy**	**AR time (years)**	**CR time (years)**	**Graft failure time (years)**	**Follow-up post transplant (years)**
Group 1												
1	31–35	3.0	P	M0E0S0T0C0	1.35	0.02	0–1	(–)	(–)	(–)	(–)	4.7
Group 2												
1	31–35	7.7	E	M1E0S0T0C0	1.07	0.83	1–4	TE	(–)	(–)	(–)	10.3
2	26–30	3.5	E	M0E0S0T0C0	1.93	0.28	1–4	TE	(–)	(–)	(–)	6.0
3	31–35	2.9	E	M1E1S1T0C1	2.10	1.85	30–49	TE/MP	(–)	(+)^*^ 2.9	(+) 9.6	9.6
4	31–35	12.1	E	M1E0S1T1C0	1.50	1.32	0–1	ARB	(–)	(+) 16.0	(+) 17.9	17.9
5	41–45	5.8	E	M1E0S1T1C1	1.40	1.26	30–49	ARB, TE/MP	(–)	(+)^*^ 5.8	(+) 9.3	9.3
6	31–35	2.3	E	M1E0S1T0C0	1.20	1.78	10–19	ARB/MP	(–)	(+)^*^ 2.3	(+) 6.3	6.3
7	31–35	7.6	E	M1E0S1T0C0	1.80	0.70	5–9	TE/MP	(–)	(–)	(–)	17.3
8	51–55	3.2	P	M0E0S0T0C0	0.89	0.03	1–4	TE	(+) 0.2	(+) 4.8	(–)	7.0
9	31–35	1.0	P	M0E0S0T0C0	1.05	0.02	0–1	TE	(–)	(–)	(–)	7.6
10	21–25	5.3	P	M0E0S0T0C0	1.62	0.09	1–4	(–)	(–)	(–)	(–)	7.7

*Bx, Kidney allograft biopsy; E, Episode biopsy; P, Protocol biopsy; TE, Tonsillectomy; MP, Methylprednisolone; ARB, Angiotensin receptor blockers; AR, Acute rejection; CR, Chronic rejection*.

* *Chronic rejection concurrent with IgA nephropathy recurrence*.

### Serum Levels of Gd-IgA1

**Figure 3:**
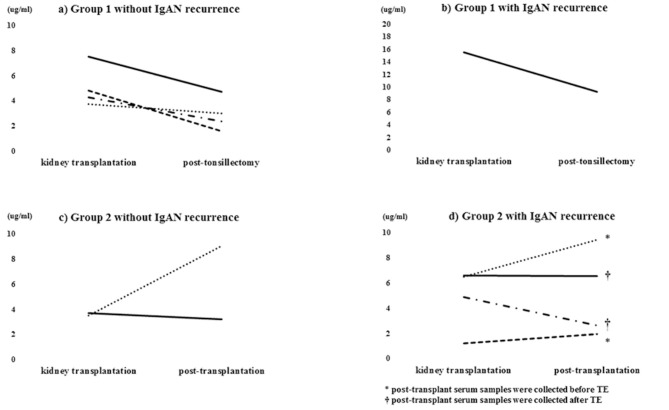
Serum Gd-IgA1 level. Serum Gd-IgA1 levels decreased after TE in group 1 **(a,b)** but remained stable or gradually increased in group 2 **(c)**. Serum Gd-IgA1 levels gradually increased before TE and decreased thereafter **(d)**.

The average serum Gd-IgA1 level at the time of kidney transplantation was 5.7 μg/ml. In group 1, serum Gd-IgA1 levels decreased after TE but the only one patient with IgAN recurrence revealed 16 μg/ml at the time of kidney transplantation and remained high 9 μg/ml level after TE (Figure 3b). In group 2, serum Gd-IgA1 levels remained stable or gradually increased and decreased after TE (Figures 3c,d). In recurrent cases in group 2, serum Gd-IgA1 levels trended to increase before TE and decrease after TE compared with the level of kidney transplantation (Figure 3d).

### Double Immunostaining for IgA and Gd-IgA1

**Figure 4:**
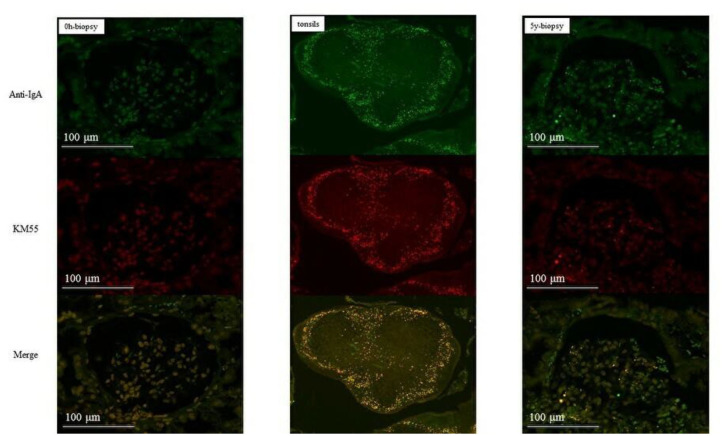
Group 1 (without IgAN recurrence). IgA and Gd-IgA1 were localized in the mantle zone of tonsils. No mesangial deposits of IgA and Gd-IgA1 were observed in the 0-h and 5-year biopsies (allograft biopsies, ×200; tonsils, ×40).

**Figure 5:**
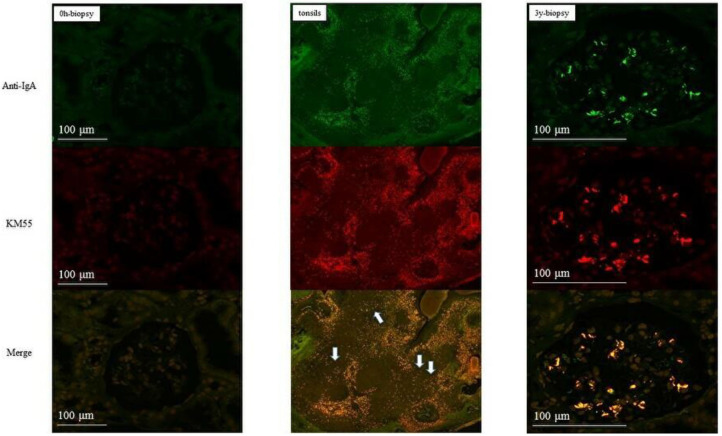
Patient 1 of group 1 (with IgAN recurrence). IgA and Gd-IgA1 deposits were observed in both the mantle zone and at the germinal center of tonsils. Mesangial deposits of IgA and Gd-IgA1 were observed in the 3-year biopsies (allograft biopsies, ×200; tonsils, ×40).

**Figure 6:**
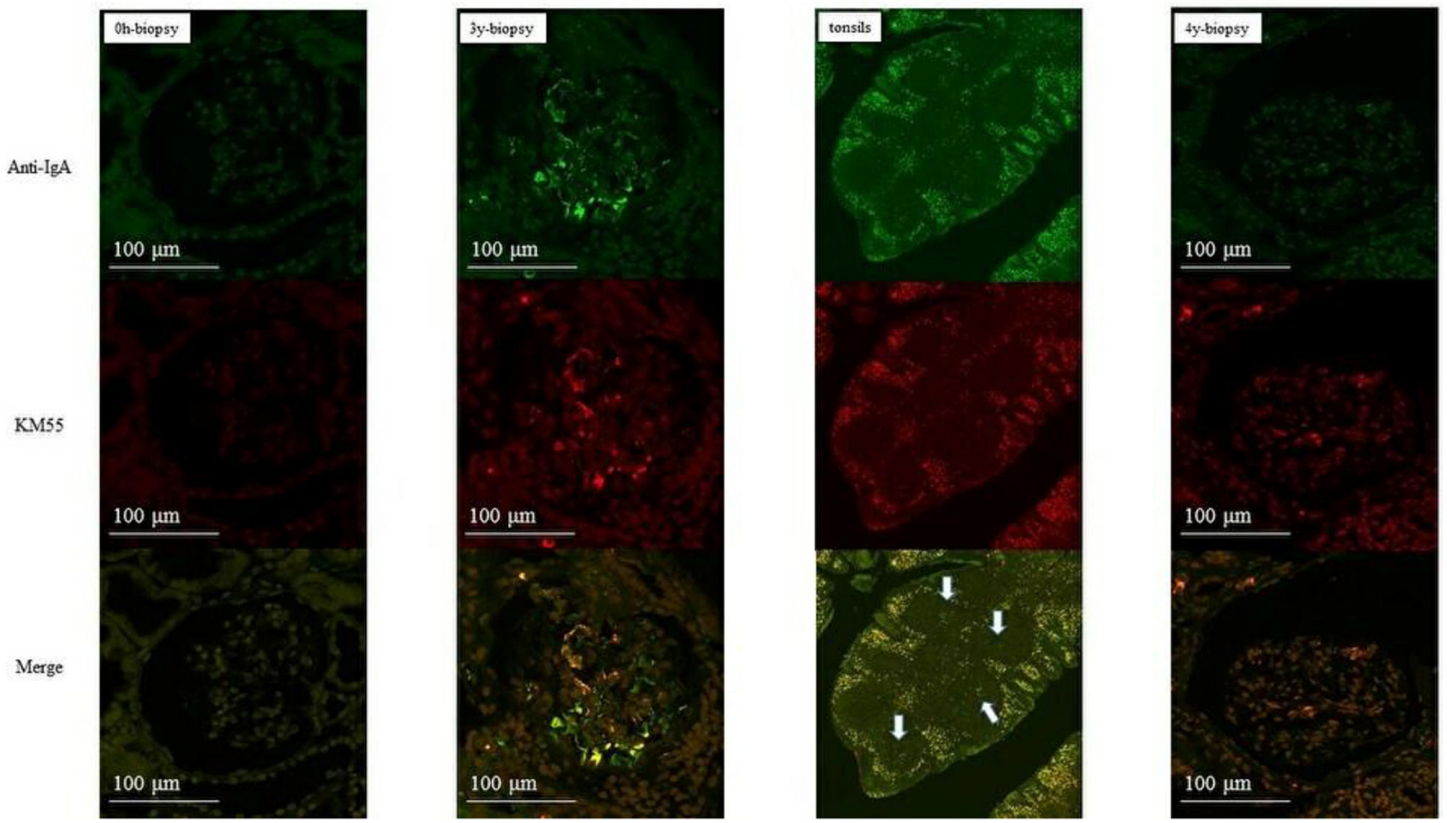
Patient eight of group 2 (with IgAN recurrence). Mesangial IgA and Gd-IgA1 deposition did not increase after TE. IgA and Gd-IgA1 were deposited at the geminal center and in the mantle zone of tonsils (allograft biopsies, ×200; tonsils, ×40).

In group 1, IgA and Gd-IgA1 deposits were observed in the mantle zone of tonsils, but no mesangial deposition was observed in the 0-h and 5-year protocol biopsies of eight of nine patients without histological IgAN recurrence (Figure 4). Mesangial IgA and Gd-IgA1 deposits were observed in the 3-year protocol biopsy of the only patient with IgAN recurrence. In tonsils, IgA and Gd-IgA1 localized predominantly in the mantle zone, but also at the germinal center (Figure 5). In patient 8 of group 2 (Table 3), the use of TE to treat 3-year histological recurrence was associated with reduced mesangial IgA and Gd-IgA1 deposition in the 4-year allograft biopsy (Figure 6). In the tonsils, IgA and Gd-IgA1 deposits were also observed at the germinal center and in the mantle zone.

**Figure 7:**
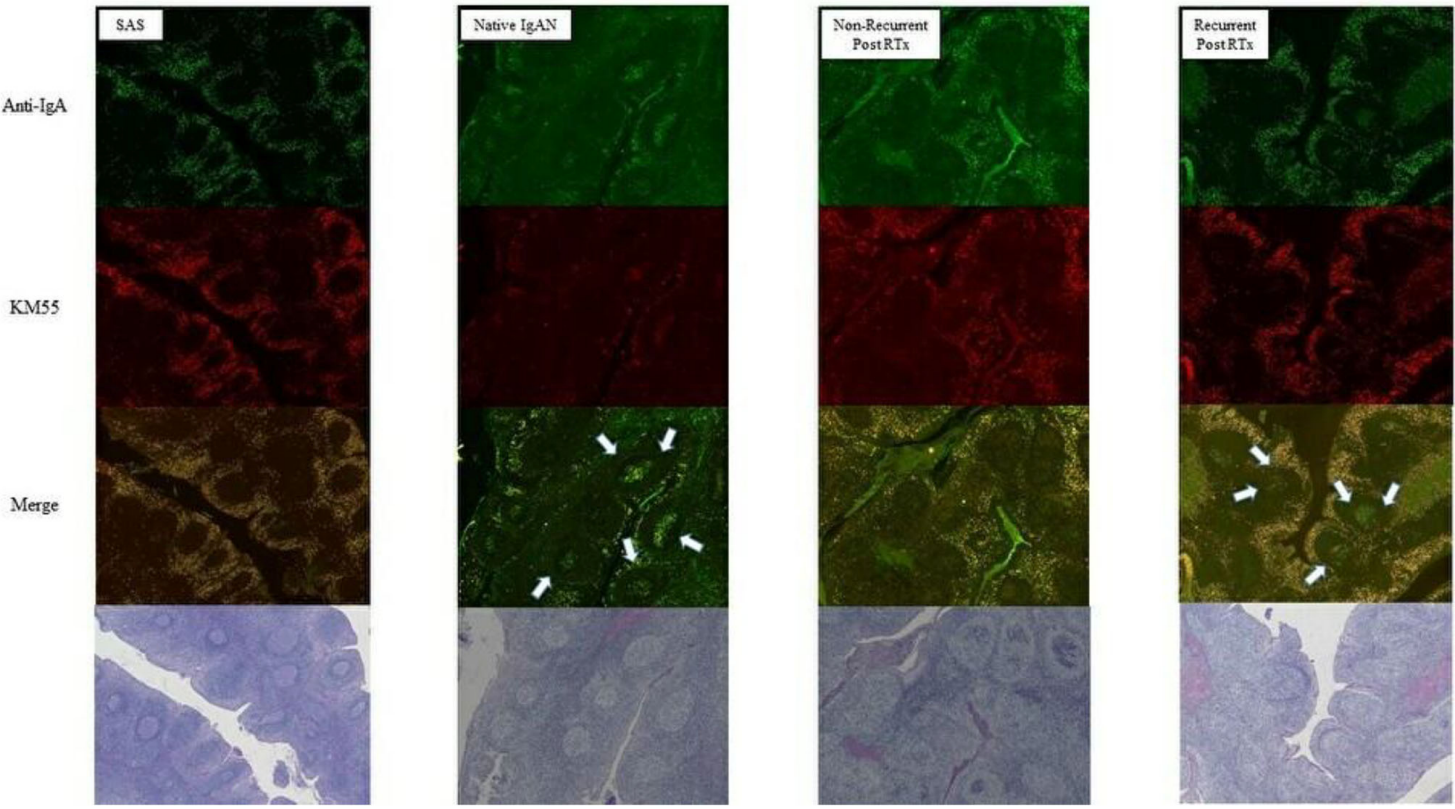
Comparison of tonsils from patients with sleep apnoea syndrome (SAS) and native IgAN. Deposition of IgA and Gd-IgA1 at the germinal center was greater in recurrent cases (magnification, ×40).

Compared with SAS and native IgAN patients, transplanted IgAN patients exhibited more severe inflammation (Figure 7). In addition, greater IgA and Gd-IgA1 deposition was observed in the mantle zone of transplanted IgAN patients than in that of native IgAN patients. Interestingly, in active cases (IgAN recurrence after transplantation or native IgAN), a central oblong immunoreactive area indicative of IgA and Gd-IgA1 was evident at the germinal center (Figure 7).

## Discussion

Prevention for recurrence of IgA nephropathy (IgAN) in the transplanted kidney are issues to be addressed. Induction therapy using anti-thymocyte globulin or steroid maintenance therapy is reported to prevent IgAN recurrence (12–14). These agents induce deep immunosuppression, increasing the susceptibility to infectious disease and malignancy. Therefore, strategies other than immunosuppression is needed in this field. In the present study, we evaluated the effect of TE at 1 year after kidney transplantation on the recurrence of IgAN in the Japanese population. Although several risk factors for IgAN recurrence have been described, e.g., younger age, zero-HLA mismatched live-related donor kidney, there was no significant differences between the baseline characteristics of the two groups in this study (2, 15). The rate of histological IgAN recurrence was significantly lower in group 1 than in group 2 (Figure 2; 11.1 vs. 55.6%, log-rank *p* = 0.046). Hirano et al. analyzed the recurrence of primary IgAN in the native kidneys of 101 patients who achieved <0.4 g/day proteinuria at 1 year after 6 months of steroid therapy, and demonstrated that steroid pulse therapy plus TE reduced the risk of recurrence in patients with mesangial hypercellularity (16). By contrast, only one study has evaluated TE before IgAN recurrence in kidney transplant patients, and concluded that TE does not affect IgAN recurrence (17). However, most TEs were performed before kidney transplantation in that study, so patients with IgAN caused by other factors such as gastrointestinal illness were included (18). Further prospective trial are needed to evaluate the preventive effect of TE on the recurrence of IgAN in transplanted kidneys.

TE was significantly associated with the reduction of serum Gd-IgA1 levels (Figure 3). The average serum Gd-IgA1 levels at the time of kidney transplantation was 5.7 μg/ml. Some reports presented serum Gd-IgA1 levels were 7.2 ± 4.0 μg/ml in healthy controls and 16.2 ± 9.1 μg/ml in native IgAN patients (19, 20). Based on these data, serum Gd-IgA1 levels in kidney transplantation might be suppressed by immunosuppression. In group 1, serum Gd-IgA1 levels decreased after TE but the only one patient with IgAN recurrence revealed the high level after TE. In group 2, Gd-IgA1 levels of recurrent patients remained stable or gradually increased before TE and decreased thereafter. By contrast, serum IgA level did not exhibit a relationship with TE (The data was not showed). Berthelot et al. suggested that Gd-IgA1, IgG autoantibodies, and IgA-sCD89 complexes are biomarkers of IgAN recurrence (21). These results suggested that TE reduced the serum Gd-IgA1 more specific than serum IgA.

In this study, the overall IgAN recurrence rate was 41% (11/27) at 5.0 ± 3.2 years. The histological recurrence rate was significantly higher (10/18; 55.6%) in group 2, and 7 of the 10 patients (70%) underwent TE. Although 5 of the 10 (50%) patients exhibited high histological activity, there was no graft loss due to recurrence. Histological recurrence of IgAN occurs at 1, 3, and 5 years post-transplantation in 12.5, 42.0, and 51.0% of patients, respectively (3). Graft survival in IgAN patients is reportedly better than that in non-IgAN patients, other than those with diabetic nephropathy, in the first 5 years after transplantation. However, at ~10–12 years, the rate of recurrence increases and reduces graft survival rate in IgAN patients (22, 23). Compared with previous reports, our data on IgAN recurrence rate were comparable, but we observed a lower rate of graft loss due to IgAN recurrence. These results implicated that TE could be an effective treatment for IgAN recurrence. Transplant patients receive three immunosuppressive therapies, so to prevent severe infections, a treatment that does not add to immunosuppression should be selected. Several studies have evaluated the use of TE for the treatment of native IgAN or IgAN recurrence. For example, Hotta et al. reported that TE and steroid pulse therapy induced clinical remission and no progressive deterioration in the native kidney in a retrospective investigation of 329 patients (24). Xie et al. reported that TE had a significant effect on kidney outcome after an average of 20 years (25). Recently, in a multicenter randomized controlled trial, Kawamura et al. showed that TE combined with steroid pulse therapy had a greater anti-proteinuric effect than a steroid pulse alone during a 12-month follow-up (26). In addition, a nationwide retrospective cohort study in Japan revealed that TE is associated with a lower risk of adverse kidney outcomes in patients with native IgAN nephropathy (27). TE is also reported to be useful for treating kidney transplant patients with IgAN recurrence. In 2009, Kennoki et al. evaluated 28 patients with IgAN recurrence and persistent proteinuria of 300 mg/day and found that TE immediately reduced the severity of proteinuria from 880 ± 630 to 280 ± 220 mg/day (*p* < 0.01), whereas little change was noted in the control group (28). Furthermore, six of seven patients experiencing recurrence underwent TE and subsequently exhibited clinical remission (29). Thus, TE may be a novel and effective treatment for IgAN recurrence, particularly at the early stages.

Acute tonsillitis can aggravate IgAN; therefore, the tonsils likely produce the Gd-IgA1 that causes IgAN (30). Double immunostaining showed that the distributions of IgA and Gd-IgA1 were identical in the tonsils and glomeruli, but of the two, serum Gd-IgA1 levels were more representative of the status of IgAN progression. IgA and Gd-IgA1 deposits were observed in the mantle zone of most tonsils; in recurrent cases in both groups, a central oblong-shaped area that was immunoreactive for IgA and Gd-IgA1 was observed at the germinal center in addition to deposits in the mantle zone. Interestingly, in patient 8, TE for a 3-year histological recurrence led to reduced mesangial IgA and Gd-IgA1 deposition in the 4-year allograft biopsy and markedly reduced Gd-IgA1 levels. Thus, serum Gd-IgA1 derived from the central oblong lesion may be key for IgAN recurrence.

This study had several limitations. First, the sample size may be small for detecting risks of recurrence. In fact, the main result was marginally significant. Therefore, results obtained in this study require further testing, by randomized clinical trials. Second, a single-center retrospective aspect may introduce selection bias and reduced the statistical power of the study. Third, the study duration was insufficient to reach firm conclusions. IgAN recurrence after transplantation can be clinically silent for many years, so a follow-up period of at least 15 years would be required to evaluate the effect of TE on graft survival. Besides these limitations, we were able to hypothesize the association of the serum Gd-IgA1 and the IgAN recurrence in kidney transplantation. In conclusion, TE at 1 year after kidney transplantation was associated with the reduction of histological IgAN recurrence rate, serum Gd-IgA1 and mesangial Gd-IgA1 immunoreactivity after transplantation. Therefore, serum Gd-IgA1 derived from the tonsils may play a pivotal role in the pathogenesis of IgAN, but further research is required to verify this finding in a prospective randomized controlled trial.

## Data Availability Statement

All datasets presented in this study are included in the article/supplementary material.

## Ethics Statement

The studies involving human participants were reviewed and approved by the ethics committee of Jikei University School of Medicine. IRB approval number: 31-226(9725), 31-278(9777). The patients/participants provided their written informed consent to participate in this study.

## Author Contributions

MK, IY, and HYamam participated in the clinical practice, designed the study protocol, and drafted the manuscript. TYa, HKa, AKa, YN, AKo, KK, HU, YT, IO, and NT participated in the patient's care and revised the manuscript. YK, JM, HYamad, and TK performed the kidney transplantation. NI and HKo performed tonsillectomy. TYo is the divisional directors and supervised each author. All authors contributed to the preparation of the manuscript and approved the final version.

## Conflict of Interest

The authors declare that the research was conducted in the absence of any commercial or financial relationships that could be construed as a potential conflict of interest.
